# *Prochlorococcus* and *Synechococcus* have Evolved Different Adaptive Mechanisms to Cope with Light and UV Stress

**DOI:** 10.3389/fmicb.2012.00285

**Published:** 2012-08-08

**Authors:** Daniella Mella-Flores, Christophe Six, Morgane Ratin, Frédéric Partensky, Christophe Boutte, Gildas Le Corguillé, Dominique Marie, Nicolas Blot, Priscillia Gourvil, Christian Kolowrat, Laurence Garczarek

**Affiliations:** ^1^Station Biologique, UPMC-Université Paris VIRoscoff, France; ^2^Groupe Plancton Océanique, CNRS, UMR 7144Roscoff, France; ^3^Departamento de Ecología, Facultad de Ciencias Biologicas, Pontificia Universidad Catolica de ChileSantiago, Chile; ^4^CNRS, FR 2424, Service Informatique et GénomiqueRoscoff, France; ^5^Laboratoire Microorganismes: Génome et Environnement, Clermont Université, Université Blaise PascalClermont-Ferrand, France; ^6^Laboratoire Microorganismes: Génome et Environnement, CNRS, UMR 6023Aubière, France; ^7^Center for Doctoral Studies, University of ViennaVienna, Austria

**Keywords:** marine cyanobacteria, *Synechococcus*, *Prochlorococcus*, light/dark cycle, light stress, UV radiations, oxidative stress, photophysiology

## Abstract

*Prochlorococcus* and *Synechococcus*, which numerically dominate vast oceanic areas, are the two most abundant oxygenic phototrophs on Earth. Although they require solar energy for photosynthesis, excess light and associated high UV radiations can induce high levels of oxidative stress that may have deleterious effects on their growth and productivity. Here, we compared the photophysiologies of the model strains *Prochlorococcus marinus* PCC 9511 and *Synechococcus* sp. WH7803 grown under a bell-shaped light/dark cycle of high visible light supplemented or not with UV. *Prochlorococcus* exhibited a higher sensitivity to photoinactivation than *Synechococcus* under both conditions, as shown by a larger drop of photosystem II (PSII) quantum yield at noon and different diel patterns of the D1 protein pool. In the presence of UV, the PSII repair rate was significantly depressed at noon in *Prochlorococcus* compared to *Synechococcus*. Additionally, *Prochlorococcus* was more sensitive than *Synechococcus* to oxidative stress, as shown by the different degrees of PSII photoinactivation after addition of hydrogen peroxide. A transcriptional analysis also revealed dramatic discrepancies between the two organisms in the diel expression patterns of several genes involved notably in the biosynthesis and/or repair of photosystems, light-harvesting complexes, CO_2_ fixation as well as protection mechanisms against light, UV, and oxidative stress, which likely translate profound differences in their light-controlled regulation. Altogether our results suggest that while *Synechococcus* has developed efficient ways to cope with light and UV stress, *Prochlorococcus* cells seemingly survive stressful hours of the day by launching a minimal set of protection mechanisms and by temporarily bringing down several key metabolic processes. This study provides unprecedented insights into understanding the distinct depth distributions and dynamics of these two picocyanobacteria in the field.

## Introduction

Phytoplanktonic cells, and in particular cyanobacteria, experience dramatic daily fluctuations of solar radiations, which can become suboptimal for photosynthetic processes around midday. Photosystem II (PSII) is particularly sensitive to these changes in photon fluxes and under unfavorable or stressful conditions its activity can decline more rapidly than most other physiological processes (Berry and Björkman, 1980; Demmig-Adams and Adams, 1992; Aro et al., 1993; Andersson and Aro, 2001). Photodamages to PSII are thought to start by the inactivation of the oxygen-evolving complex, which is caused by the dissociation of the Mn_4_Ca^2+^ cluster. This process leads to the production of long-lived P_680_^+^, the oxidized form of the reaction center chlorophyll (Chl) pair, a particularly strong oxidant which in turn provokes the destruction of the PSII core protein D1 (Hakala et al., 2006; Nishiyama, 2006). At low irradiances, the rate of photosynthetic electron transport is proportional to the photon flux density and damaged D1 polypeptides can be removed from the PSII reaction center and rapidly replaced by newly synthesized D1 proteins (Park et al., 1995; Tyystjarvi and Aro, 1996; Nixon et al., 2005; Ohnishi et al., 2005). However, at higher irradiances, the rate at which the PSII reaction center is damaged can exceed its repair rate, which results in an increase of inactivated PSII centers and a subsequent decline of the quantum yield of photosynthesis, resulting from photoinhibitory fluorescence quenching (Powles, 1984; Prásil et al., 1992; Aro et al., 1993; Andersson and Aro, 2001).

Although the visible part of the solar spectrum (400–700 nm), also called photosynthetically active radiations (PAR), is responsible for most photoinhibitory effects, the contribution of UV-B (280–315 nm) and, to a least extent, UV-A (315–400 nm) is also notable in the uppermost layer of the ocean (Dring et al., 2001; van de Poll et al., 2001; He and Häder, 2002b). UV-B can indeed damage the photosynthetic apparatus about 100-fold more efficiently than visible light and these radiations might directly affect PSII proteins and the Mn_4_Ca^2+^ cluster (Sarvikas et al., 2006; Caldwell et al., 2007). UV and high visible radiations can also cause indirect photoinhibitory effects via the production of reactive oxygen species (ROS; He and Häder, 2002a,b; Rastogi et al., 2010), mainly formed within reaction centers (Asada, 1999) and light-harvesting complexes (Knox and Dodge, 1985; Zolla and Rinalducci, 2002). ROS are powerful oxidizing agents which can react with DNA, lipids, and proteins. Although these compounds are inevitably produced by cell metabolism, even under optimal growth conditions, their production is drastically enhanced when cells are exposed to a variety of stresses, including excess visible light and UV radiations (UVR; Latifi et al., 2005; Ross et al., 2006; Houot et al., 2007; Allakhverdiev and Murata, 2008). The effect of ROS on PSII photoinhibition is thought to act primarily by inhibiting the *de novo* synthesis of proteins, including those required for the repair of PSII (Nishiyama et al., 2004; Nishiyama, 2006; Takahashi and Murata, 2008). A direct effect of ROS on the inactivation of PSII reaction center has also been suggested through triggering D1 degradation (Vass et al., 1992; Aro et al., 1993; Miyao et al., 1995; Keren et al., 1997; Lupinkova and Komenda, 2004). In any case, ROS clearly have a major role in light-mediated photoinhibition as well as in other environmental stresses (Nishiyama, 2006; Allakhverdiev and Murata, 2008; Latifi et al., 2009). Thus, survival of phototrophic organisms depends upon the amount of ROS produced and their efficiency in scavenging these oxygen species.

In this context, marine picocyanobacteria belonging to the genera *Synechococcus* and *Prochlorococcus* constitute two relevant and complementary models to study acclimation processes to high light and UVR and their interrelationships with oxidative stress. In oceanic ecosystems, these two organisms numerically dominate the phytoplanktonic community (Partensky et al., 1999a; Scanlan, 2003) and are considered to be the two most abundant photosynthetic organisms on Earth, with a substantial contribution to Chl biomass and primary production (Liu et al., 1997; Partensky et al., 1999a; Agawin et al., 2000; Garcia-Pichel et al., 2003). Members of the marine *Synechococcus* genus are ubiquitously distributed and are most abundant in coastal regions and mesotrophic open ocean surface waters (Partensky et al., 1999a; Zwirglmaier et al., 2008), whereas *Prochlorococcus* preferentially thrives in warm, stratified, oligotrophic tropical, and subtropical marine areas (Partensky et al., 1999b; Zubkov et al., 2000; Johnson et al., 2006). In the field, these organisms experience large variations in irradiance, linked to the combination of the light/dark (L/D) cycle, water mixing, and a variable cloudiness (MacIntyre et al., 2000). Moreover, their tiny size (0.5–0.8 and 0.8–1.2 μm diameter for *Prochlorococcus* and *Synechococcus*, respectively) confers them a high surface to volume ratio, optimizing their photon capture, and making them particularly sensitive to UVR (Llabres and Agusti, 2006, 2010).

Like other photosynthetic organisms, marine cyanobacteria have evolved a variety of protection mechanisms to ensure their growth and survival in highly illuminated habitats. These mechanisms include thermal dissipation of excess light excitation, structural changes of the photosynthetic machinery as well as enzymatic and non-enzymatic scavenging systems to eliminate ROS, in particular those produced in photosynthetic membranes (for reviews, see Bailey and Grossman, 2008; Latifi et al., 2009). However, several pieces of evidence suggest that *Prochlorococcus* and *Synechococcus* lineages could deal differently with light stress. Indeed, two *P. marinus* strains (PCC 9511 and SS120, a high light- and a low-light-adapted ecotype, respectively) were found to be more sensitive to a transient exposure to high irradiances than three *Synechococcus* spp. strains representative of various trophic environments and exhibiting different pigmentation (RS9917, RCC307, and WH8102; Six et al., 2007b). Similarly, measurements of cell abundances and/or mortality rates of field populations of picocyanobacteria exposed to different levels of natural solar radiations showed that *Prochlorococcus* exhibited a lower resistance to UVR than *Synechococcus* in surface waters of the central Atlantic Ocean (Llabres and Agusti, 2006; Agusti and Llabres, 2007) and the Mediterranean Sea (Sommaruga et al., 2005; Llabres and Agusti, 2010).

In order to reveal potential differences in circadian metabolic rhythms between these two genera, the photophysiology of the model strains *P. marinus* PCC 9511 and *Synechococcus* sp. WH7803 was examined at different times of a modulated L/D cycle of visible light (hereafter VL) with or without UV. Additionally, the diel variability of the sensitivity of *Prochlorococcus* and *Synechococcus* to oxidative stress, as triggered by different H_2_O_2_ concentrations was investigated. Expression of key genes involved in photosynthesis, light, and oxidative stress response and a number of other processes were also monitored in order to get insights about the molecular bases of the observed physiological differences.

## Materials and Methods

### Strains and culture conditions

The two model strains *P. marinus* PCC 9511 [a strain genetically very close, if not identical, to MED4 (Rippka et al., 2000; Rocap et al., 2003)] and *Synechococcus* sp. WH7803 (Scanlan et al., 2009) used in this study were grown at 22°C in PCR-S11 medium (Rippka et al., 2000), supplemented with 1 mM NaNO_3_. All experiments were performed under modulated L/D conditions using a computer-controlled illumination device (cyclostat), as detailed elsewhere (Holtzendorff et al., 2001; Kolowrat et al., 2010). This system allows the simulation of a bell-shaped 12/12 h L/D cycle, which induces a good synchronization of cell division, as observed for field populations (Vaulot et al., 1995; Jacquet et al., 1998). The maximal VL irradiance (at virtual noon) was set at 870 μmol photons m^−2^ s^−1^, corresponding to the reference light condition. In order to test the specific effects of UVR, the same experiments were repeated but with supplementing the VL condition with modulated UV-A and UV-B radiations (hereafter VL + UV), provided by UV-A-340 fluorescent lamps (Q-Panel Lab products, Cleveland, OH, USA). UVR reached 7.59 W m^−2^ UV-A (320–340 nm) and 0.57 W m^−2^ UV-B (280–320 nm) at virtual noon, corresponding to levels representative of natural doses measured in the upper layer of nutrient-poor, oceanic areas (Helbling et al., 1992). Two replicate cultures were acclimated to L/D cycles for at least 2 weeks prior to start monitoring the different parameters. During the experiment, two replicate cultures were grown with a continuous input of fresh medium, in order to maintain cells in exponential growth throughout the whole sampling period. One microliter aliquots were taken every hour, fixed for 10 min with grade I glutaraldehyde (0.25% final concentration; Sigma Aldrich, Saint-Louis, MO, USA), then frozen at −80°C for delayed analyses of cell abundance and cell cycle using a FACS Canto flow cytometer (Becton Dickinson Biosciences, San Jose, CA, USA), as previously described (Marie et al., 1997, 1999). Because of the continuous dilution, growth rate of these cultures was indirectly assessed from cell cycle data (μ_cc_) using the method described by Carpenter and Chang (1988), as detailed earlier (Kolowrat et al., 2010). To study the kinetics of response to light fluctuations, cultures were sampled at 6, 9, 12, 15, 18, 20, 22, and 2 h over 3 days for measuring a variety of parameters described below.

### Pigment and fluorescence measurements

Photosynthetic pigments were extracted in 95% methanol and analyzed by HPLC, as previously described (Everroad et al., 2006). Whole cell fluorescence emission spectra with excitation at 530 nm were recorded for *Synechococcus* using a LS-50B spectrofluorometer (Perkin Elmer, Waltham, MA, USA). The PSII quantum yield (*F*_V_/*F*_M_) was measured using a Pulse Amplitude Modulated fluorimeter (PhytoPAM, Walz, Effeltrich, Germany) connected to a LabPro chart recorder allowing the direct visualization of fluorescence traces (Vernier, Beaverton, OR, USA). A 2 mL aliquot was dark acclimated for 5 min in a quartz cuvette with a mirrored facet facing the photomultiplier to enhance the signal. The modulated light was then turned on to measure the basal fluorescence level F_0_ with modulated excitation at 440 nm for *Prochlorococcus* cells and 520 nm for *Synechococcus*. The maximal fluorescence *F*_M_ was determined by applying a light saturating pulse in the presence of 50 μM of the PSII inhibitor 3-(30,4-dichlorophenyl)-1,1-dimethylurea (DCMU) under ca. 2,000 μmol photons m^−2^ s^−1^. The PSII quantum yield was calculated as:

(1) $$F_{\text{V}}\text{/}F_{\text{M}}=\left(F_{\text{M}}\text{ - }F_{\text{0}}\right)∕F_{\text{M}}$$

where *F*_V_ is the variable fluorescence.

### Photosystem II repair

At each sampling time point, a 20 mL volume of each replicate culture was sampled and split into two 100 mL quartz Erlenmeyer flasks, and one of them was supplemented with 500 μg mL^−1^ lincomycin, an inhibitor of protein translation (Six et al., 2007a). The two flasks were immediately brought back into the culture system and 2 mL aliquots were collected at 0, 15, 30, and 60 min after the sampling to measure the PSII quantum yield, as described above. The PSII quantum yield was then plotted over time for both the control and lincomycin-treated sub-cultures and plots were fitted with an exponential decay function. The PSII repair rate was estimated as the difference between the exponential decay rates in the absence and presence of lincomycin (Six et al., 2007a, 2009; Campbell and Tyystjarvi, 2012).

### Immunoblotting

Cell pellets were resuspended in extraction buffer (140 mM Tris base, 105 mM Tris-HCl, 0.5 mM ethylenediaminetetraacetic acid, 2% lithium dodecyl sulfate, 10% glycerol, and 0.1 mg mL^−1^ PefaBloc protease inhibitor (Roche, Basel, Switzerland) and total protein concentration was determined using a Lowry protein assay kit (Bio-Rad, Hercules, CA, USA) and bovine serum albumin as protein standards. Samples were then denatured with 50 mM dithiothreitol and heated for 2 min at 80°C and 2 μg total protein was loaded on a 4–12% gradient acrylamide precast NuPAGE Bis-Tris mini-gel (Invitrogen, Carlsbad, CA, USA) along with recombinant standards of D1 or D2 proteins (Agrisera, Vännäs, Sweden) to establish a standard curve. Gels were electrophoresed and the proteins were transferred onto a polyvinylidene fluoride (PVDF) membrane, then immediately immersed into Tris Buffer Saline-Tween (TBS-T) buffer, pH 7.6 (0.1% Tween 20, 350 mM sodium chloride, 20 mM Trizma base) containing 2% (w:v) ECL Advance blocking agent (Amersham Biosciences, Piscataway, NJ, USA). Aliquots of primary antibodies against D1 or D2 proteins (Agrisera) were diluted at 1:50 000 in TBS-T in the presence of 2% blocking agent and membranes were soaked into this solution for 1 h with agitation. After extensive washing of the membrane with TBS-T buffer, anti-rabbit secondary antibodies were applied with the same procedure as for primary antibodies. Membranes were developed by chemoluminescence using the ECL Advance reagent kit (Amersham Biosciences) and visualized with a LAS4000 imager equipped with a CCD camera (GE Healthcare, Waukesha, WI, USA). Signals were quantified using the ImageQuant software and the recombinant protein standard curve. To ease comparisons of the diel trends between strains and conditions, data were normalized at 6:00 am.

### Oxidative stress assays

Samples collected from dawn to dusk (6, 9, 12, 15, and 18 h time points) were split into aliquots of 2.5 mL and subjected to a series of H_2_O_2_ concentrations ranging from 0.1 to 1,800 μM. These culture aliquots were then incubated in the culturing system for 50 min and the PSII quantum yield was measured as described above. To quantitatively estimate the PSII resistance to such oxidative stress at each time point, the decay of *F*_V_/*F*_M_ as a function of increasing H_2_O_2_ concentration was plotted as a percentage of *F*_V_/*F*_M_ of the control cultures. The decay curves were then fitted with a two-parameter hyperbolic decay function:

(2) $$y=ab∕\left(b+x\right)$$

The *b*-value proved to be informative because it integrates both the minimal H_2_O_2_ concentration needed to cause a decrease of *F*_V_/*F*_M_ and the decrease rate of the PSII quantum yield. Thus, this parameter can be used as a proxy to assess the global resistance of PSII to oxidative stress induced by H_2_O_2_.

### RNA extraction and real time quantitative PCR

Samples for RNA extraction were harvested and extracted as previously described in Kolowrat et al. (2010) for eight data points per L/D cycle and for two different days, corresponding to biological replicates. Briefly, cell pellets, resuspended in Trizol (Invitrogen, Carlsbad, CA, USA), were extracted using the miRNeasy kit as recommended by the manufacturers (Qiagen, Valencia, CA, USA), followed by two successive DNase treatments performed on the miRNeasy columns using the Qiagen RNase-free DNase Set (Qiagen).

Real time quantitative PCR (hereafter qPCR) was performed on a set of *P. marinus* PCC 9511 and *Synechococcus* sp. WH7803 genes, representative of key metabolic processes (Table S1 in Supplementary Material). Design and optimization of gene specific primers were performed as previously described (Six et al., 2007b) using PrimerExpress™ software v2.0 (Applied Biosystems) and by checking for every set of primers, the linearity of the CT (cycle at threshold) vs. cDNA content within a dilution range of cDNA. Reverse transcription was carried out on 100 ng RNA using SuperScriptII reverse transcriptase (Gibco-BRL, Gaithersburg, MD, USA). qPCR was performed in triplicate on the cDNA obtained after dilution, using the DNA Engine/Chromo4 Real Time PCR-Detector (Bio-Rad, Hercules, CA, USA) and the absolute SYBR Green ROX Mix (Abgene, Epsom, UK), as previously described (Garczarek et al., 2008). Gene expression profiles monitored during L/D cycles were expressed as the ratio of gene expression vs. expression of the aperiodic gene *rnpB* (Mary and Vaulot, 2003; Zinser et al., 2009; Kolowrat et al., 2010) and normalized to the 6:00 time point sampled under VL conditions, using the 2^−ΔΔCT^ method (Schmittgen and Livak, 2008).

## Results

### Cell cycle

**Figure 1:**
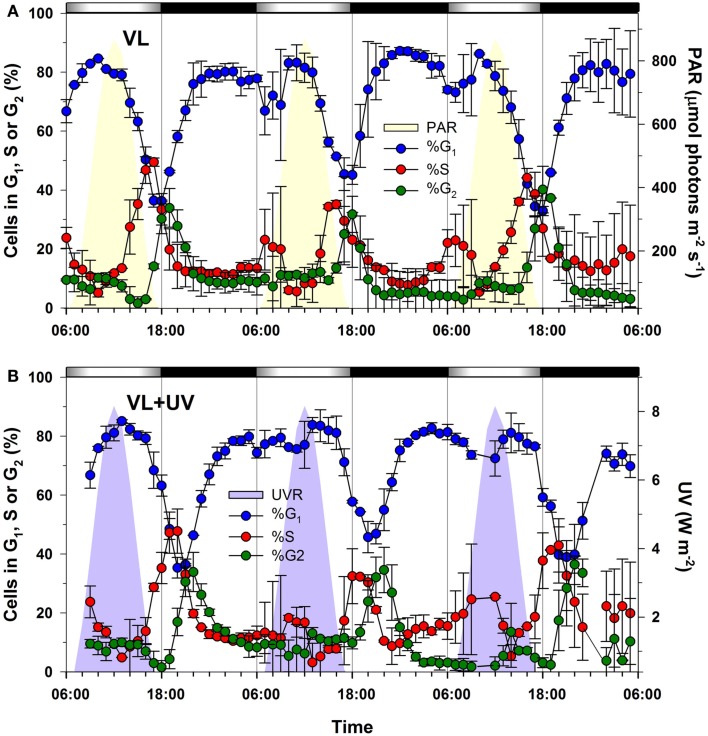
**Effect of UV exposure on the timing of the cell cycle phases of *Synechococcus* sp. WH7803 cells grown over a modulated 12/12 h L/D cycle with or without UV radiations**. **(A)** Distribution of G_1_ (blue), S (red), and G_2_ (green) phases for cells acclimated to VL. **(B)** Same for VL + UV conditions. Error bars indicate mean deviation for two biological replicates. Note that only the total UVR (UV-A + UV-B) plot is shown in graph **(B)** since PAR was the same as in graph **(A)**. White and black bars above graphs indicate light and dark periods, also delineated by gray vertical bars and areas filled in yellow (PAR) or purple (UV). Abbreviations: L/D, light/dark; PAR, photosynthetically available radiations; VL, visible light; UV, ultraviolet.

The cell cycle of *Synechococcus* sp. WH7803 was strongly synchronized by the alternation of light and darkness, with a peak of DNA replicating cells (S cells) occurring at ca. 16–17:00, i.e., 1–2 h before the light-to-dark transition (LDT; 18:00), in modulated VL only and about 3 h later for cells acclimated to modulated VL + UV (Figure 1). A comparable delay of the S phase was previously reported for *P. marinus* PCC 9511 in the presence of UVR, but the peak of S cells occurred at the LDT in VL and about at the same time as for *Synechococcus* in VL + UV (Kolowrat et al., 2010). Another notable difference with *Prochlorococcus* is the occurrence of a second minor S peak in the early morning followed by a small bump of G_2_ cell abundance around noontime (mainly visible during the first and third cycle), suggesting some ultradian growth (Shalapyonok et al., 1998). Mean growth rates of *Synechococcus* cultures were assessed from the percentages of cells in S and G_2_ (μ_cc_) using the method described by Carpenter and Chang (1988). They were not statistically distinct between the two conditions (average over 3 days and two replicates: μ_cc_ = 1.00 ± 0.21 day^−1^ in VL; μ_cc_ = 1.23 ± 0.11 day^−1^ in VL + UV) but were higher than in *Prochlorococcus* (μ_cc_ = 0.67 ± 0.05 day^−1^ in VL; μ_cc_ = 0.68 ± 0.03 day^−1^ in VL + UV; Kolowrat et al., 2010). This difference is attributable in part to the shorter delay observed between the maxima of S and G_2_ cells in *Synechococcus* (t_G2_ − t_S_ ∼ 2.5 h) than in *Prochlorococcus* (t_G2_ − t_S_ ∼ 4 h), as this parameter is used for the calculation of growth rates in the μ_cc_ method (Carpenter and Chang, 1988).

### Pigment ratios

**Figure 2:**
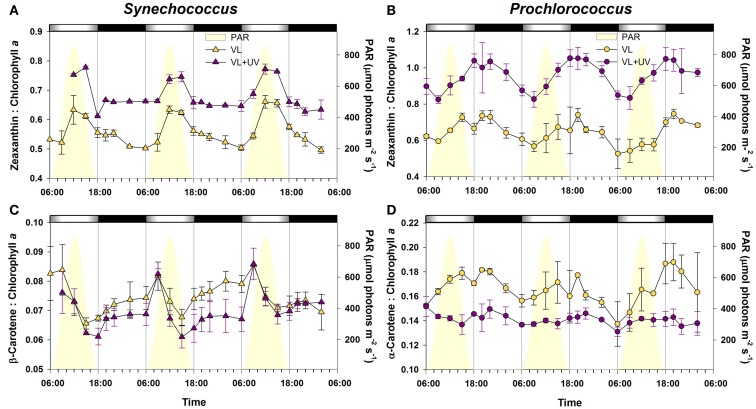
**Daily variations of the ratios of the two major carotenoids (zeaxanthin and carotene) to chlorophyll *a* for picocyanobacterial cells acclimated to a modulated 12/12 h L/D cycle of VL with or without UV radiations**. **(A,C)** *Synechococcus* sp. WH7803. **(B,D)** *Prochlorococcus marinus* PCC 9511. White and black bars above graphs indicate light and dark periods, also delineated by vertical bars and areas filled in yellow. Error bars indicate mean deviation for two biological replicates. Abbreviations as in Figure 1.

Comparative diel variations of molar ratios of the main pigments in the two marine picocyanobacteria under both light conditions are reported in Figure 2. For *Synechococcus* grown under VL, the zeaxanthin (Zea) to Chl *a* ratio (Figure 2A) was maximal between noon and the early afternoon, and then decreased sharply till the LDT and at a lower rate during the night. In VL + UV, the pattern was globally similar, except that (i) the values were higher in VL + UV than in VL, (ii) the midday peak had a lower average amplitude (15.4 vs. 25.4% increase under VL + UV and VL, respectively), and (iii) the ratio remained stable during the night. By comparison, the β-carotene (β-Car) to Chl *a* ratio of *Synechococcus* grown in VL systematically peaked at 9:00, decreased till 15:00 then increased again for the rest of the diel cycle (Figure 2C). The pattern was very similar under UV, except that night values were slightly lower during the first two L/D cycles. The pattern of pigment ratios observed in *Prochlorococcus* exhibited a number of differences compared to *Synechococcus*. In VL, the Zea to divinyl-(DV-) Chl *a* ratio started to increase at mid-morning and was maximal around 20:00, then decreased during the rest of the night (Figure 2B), corresponding to the cell division phase. The same diel pattern was observed under VL + UV. Although the variation range was moderate for the α-Car to DV-Chl *a* ratio in VL, its diel pattern was comparable to that of the Zea to DV-Chl *a*, except that it increased immediately after dawn (Figure 2D). There were almost no diel oscillations of this ratio under VL + UV. The DV-Chl *b* to *a* ratio exhibited no clear diel pattern in either light condition (data not shown).

### Photosystem II function and repair

**Figure 3:**
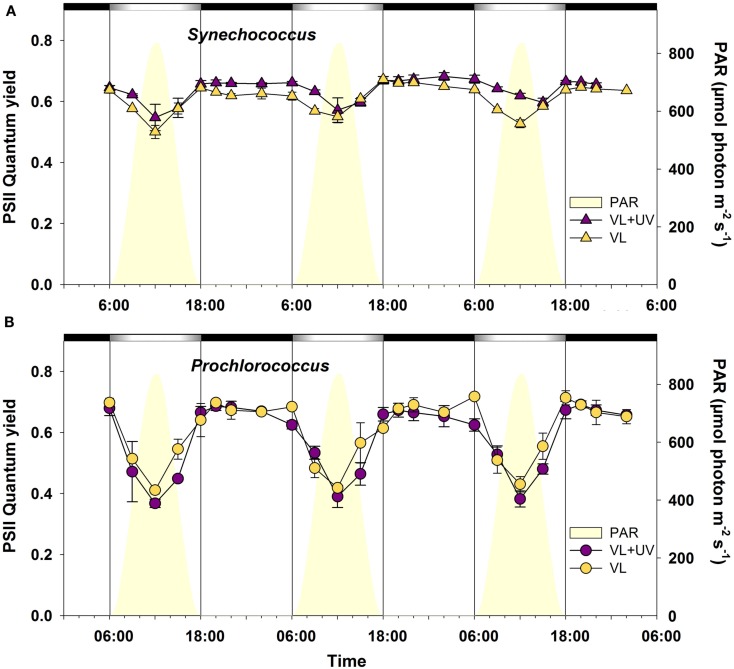
**Daily variations of the photosystem II maximal quantum yield (*F*_V_/*F*_M_) for picocyanobacterial cells acclimated to a modulated 12/12 h L/D cycle of VL with or without UV radiations**. **(A)** *Synechococcus* sp. WH7803, **(B)** *Prochlorococcus marinus* PCC 9511. White and black bars above graphs indicate light and dark periods, also delineated by vertical bars and areas filled in yellow. Error bars indicate mean deviation for two biological replicates. Abbreviations as in Figure 1.

In order to investigate how the daily variations of VL and UVR could affect the PSII activity of cells, we followed the PSII quantum yield in *Synechococcus* sp. WH7803 and *P. marinus* PCC 9511 cultures during three consecutive L/D cycles (Figure 3). For both strains and under both light conditions, the *F*_V_/*F*_M_ ratio showed a cyclic evolution, reaching a maximum at night and a minimum at virtual noon. This midday drop, originating either from PSII photoinactivation or from dissipative non-photochemical quenching (NPQ) of fluorescence (or both), was larger for *Prochlorococcus* than for *Synechococcus* under both light conditions. Indeed, there was a relative decrease of the PSII quantum yield of about 40% for *Prochlorococcus*, while in *Synechococcus* it never exceeded 20% (Figure 3). The diel patterns of PSII activities showed also some differences between VL and VL + UV, with slightly higher late night and morning yields for *Synechococcus* and lower yields at 6:00, 12:00, and 15:00 for *Prochlorococcus* cells in the latter condition.

**Figure 4:**
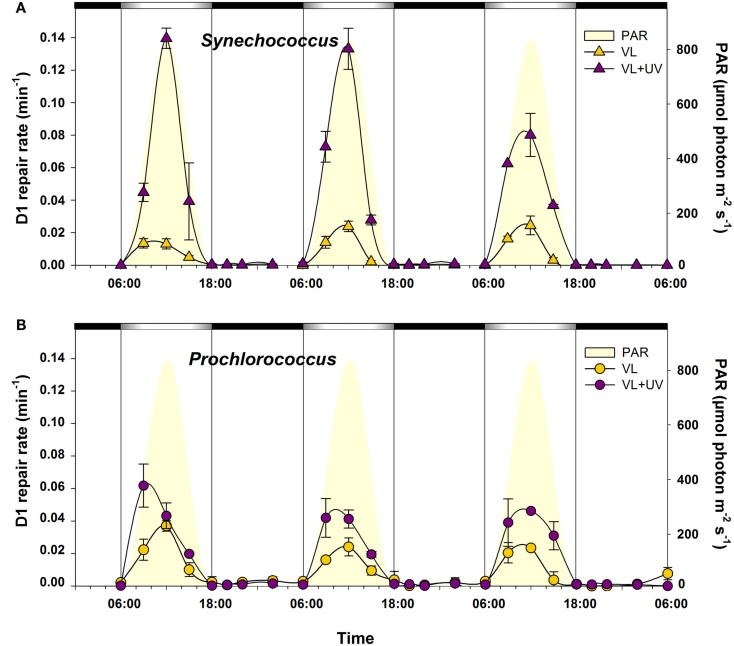
**Daily variations of the photosystem II repair activity for picocyanobacterial cells acclimated to a modulated 12/12 h L/D cycle of VL with or without UV radiations**. **(A)** *Synechococcus* sp. WH7803, **(B)** *Prochlorococcus marinus* PCC 9511. White and black bars above graphs indicate light and dark periods, also delineated by vertical bars and areas filled in yellow. Error bars indicate mean deviation for two biological replicates. Abbreviations as in Figure 1.

Variations in the PSII repair activity of the cells, as assessed by comparing the PSII quantum yield of sub-cultures incubated with or without lincomycin, an inhibitor of protein synthesis, also showed a cyclic pattern, with no measurable PSII repair occurring during the night (Figure 4). Moreover, cells acclimated to VL and VL + UV conditions exhibited a very different repair rate, especially in *Synechococcus*. While VL exposure only led to a moderate induction of the PSII repair rate with regard to night levels in *Synechococcus*, a sharp increase of this rate (from three- to six-fold) was observed for cells acclimated to UV-supplemented light (Figure 4A). Such an UV-induced increase of the daily PSII repair rate was also observed in *Prochlorococcus* cells, although much more limited (less than twofold), and the maximal rate were already reached at 9:00 (Figure 4B).

### Photosystem II core protein pools

**Figure 5:**
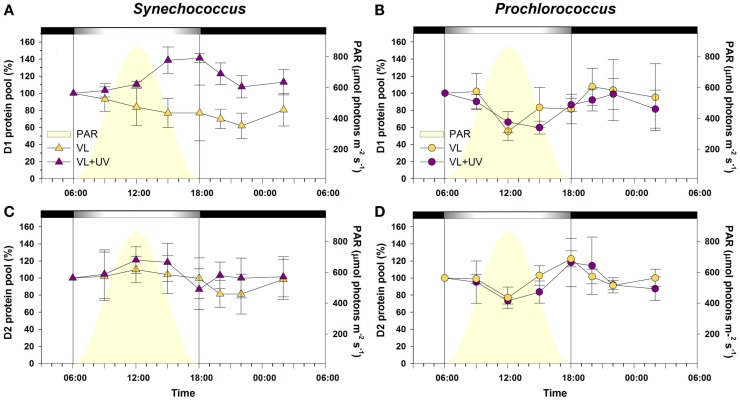
**Daily variations of the total pools of photosystem II core proteins D1 and D2 for picocyanobacterial cells acclimated to a modulated 12/12 h L/D cycle of high visible light with or without UV radiations**. **(A,C)** *Synechococcus* sp. WH7803, **(B,D)** *Prochlorococcus marinus* PCC 9511. White and black bars above graphs indicate light and dark periods, also delineated by vertical bars and areas filled in yellow. Data represent the mean ± standard deviation (*n* = 3–4) of two biological replicates and two consecutive days. Abbreviations as in Figure 1.

In *Synechococcus* cells grown under VL, both the D1 and D2 protein pools showed no significant diel oscillations during the L/D cycle (Figure 5). Under VL + UV, while D2 also remained stable, cells progressively accumulated D1 proteins during daytime and the pool reached a maximum at the LDT (40% increase compared to the value at 6:00). *Prochlorococcus* cells showed a quite different diel pattern of D1 and D2 protein pools. Under VL, both proteins showed minimal contents during the day (30–40% decrease), when irradiance was maximal, with a subsequent full recovery during the night. A similar pattern was observed under VL + UV, but with an extended period of low PSII core protein content.

### Resistance of PSII to oxidative stress

**Figure 6:**
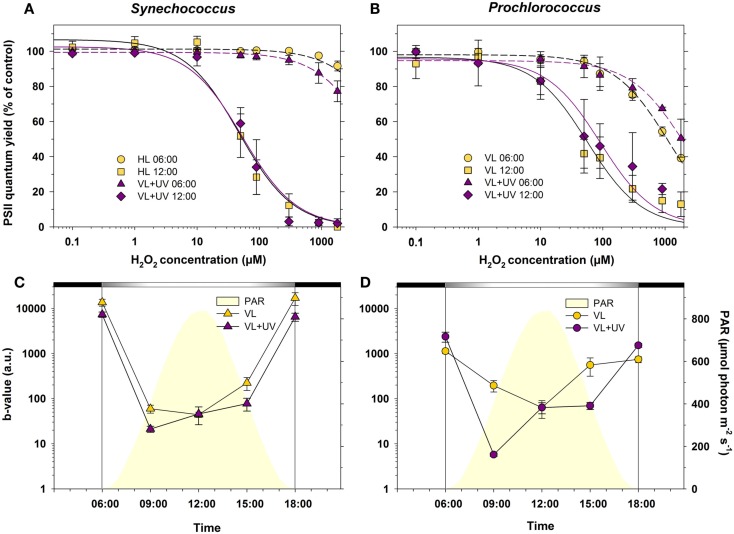
**Daily variations of photosystem II (PSII) resistance to oxidative stress for picocyanobacterial cells acclimated to a modulated 12/12 h L/D cycle of high visible light with or without UV radiations**. **(A,B)** Maximal PSII quantum yield (*F*_V_/*F*_M_) measured at 06:00 and at noon, after 50 min exposure to a range of H_2_O_2_ concentrations of *Synechococcus* sp. WH7803 **(A)** or *Prochlorococcus marinus* PCC 9511 cells **(B)**. Data were fitted with a two-parameter hyperbolic decay function. **(C,D)** PSII resistance to oxidative stress in *Synechococcus* **(C)** and *Prochlorococcus* **(D)** cells, as estimated by the *b*-value (see Materials and Methods). White and black bars above graphs **(C,D)** indicate light and dark periods, also delineated by vertical bars and areas filled in yellow. Data represent the mean ± standard deviation (*n* = 4) of two biological replicates and two consecutive days. Abbreviations as in Figure 1.

*Synechococcus* and *Prochlorococcus* grown under VL and VL + UV conditions exhibited quite different PSII responses to oxidative stress (Figure 6). *Synechococcus* sub-cultures sampled at 6:00 (i.e., at the dark-to-light transition) showed no significant change in their PSII quantum yield until H_2_O_2_ concentrations as high as 300 and 1,800 μM in VL + UV and VL, respectively. Sub-cultures sampled at 12:00 were affected by concentrations as low as 10 and 50 μM H_2_O_2_, the photoinhibitory effect increasing with the concentration of oxidizing agent until a total shutdown of PSII activity at 300 and 900 μM H_2_O_2_ for VL + UV and VL, respectively (Figure 6A). PSII inactivation occurred at lower doses in *Prochlorococcus* at both time points, starting at about 100 μM H_2_O_2_ for cultures collected at 6:00 and reaching 60 and 50% of photoinhibition at 1,800 μM H_2_O_2_ in VL and VL + UV, respectively (Figure 6B), compared to less than 20% for *Synechococcus* cells at the same time point. Furthermore, as for *Synechococcus*, *Prochlorococcus* sub-cultures sampled at noon were sensitive to lower H_2_O_2_ concentrations than cultures sampled at 6:00.

In order to compare the rates of decay of the PSII quantum yield obtained at various times of the day, *F*_V_/*F*_M_ vs. H_2_O_2_ concentration curves for all time points (only those obtained for 6:00 and 12:00 are shown in Figures 6A,B) were fitted with a two-parameter hyperbolic decay function. The daily variations of the *b*-value, characterizing the global resistance of PSII to oxidative stress induced by H_2_O_2_ (see Materials and Methods), are shown in Figures 6C,D. As expected from results at 6:00 and 12:00 (Figures 6A,B), PSII proved to be much more sensitive to an artificially induced oxidative stress during the day than during the night in both picocyanobacteria. Still, *Synechococcus* PSII was seemingly much more resistant than *Prochlorococcus* to this stress, when applied in the dark. It is also worth noting that the sensitivity to oxidative stress was enhanced for UV-acclimated cells, especially in *Prochlorococcus*, which displayed a dramatic drop of the *b*-value at 09:00 compared to those grown in VL only. However, after this sharp mid-morning drop, the *b*-value rose again during the day in both strains.

### Transcriptomic response

#### Photosynthesis

**Figure 7:**
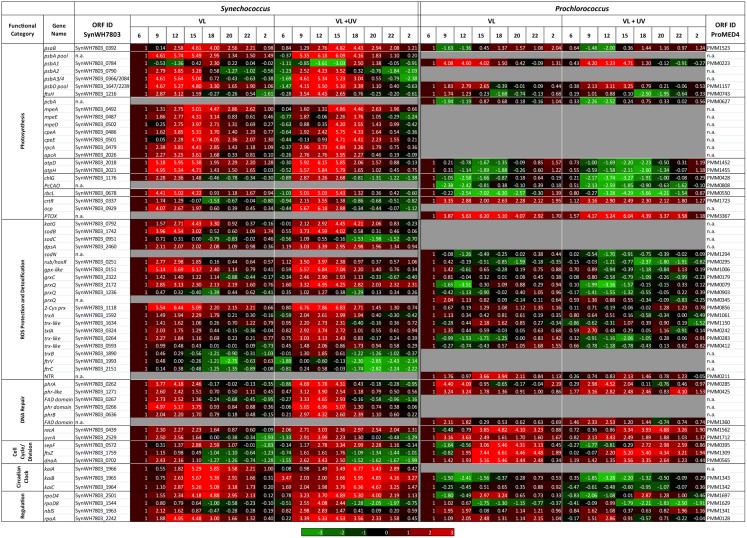
**Daily variations of the relative expression of selected genes, as measured by quantitative real time PCR, for picocyanobacterial cells acclimated to a modulated 12/12 h L/D cycle of high visible light with or without UV radiations**. All data, expressed as log_2_ (Fold Change), were normalized to the value observed at 06:00 in the VL condition for each strain and are shown as a range of red (relative upregulation) or green (relative downregulation) colors, with intensity depending on gene expression levels, as indicated in the scale bar below the data. Genes are classified by functional categories, as indicated in the first column. A more detailed listing of these data showing mean deviation for two biological replicates as well as gene products is provided as Table S2 in Supplementary Material.

The diel expression pattern of a selection of genes involved in photosynthesis and a number of other processes related to (or affected by) light or UV stress were analyzed by qPCR in *P. marinus* PCC 9511 and *Synechococcus* sp. WH7803 strains, grown under both VL and VL + UV conditions (Figure 7). As expected, photosynthetic genes were among those showing the strongest diel oscillations. In both strains, the daily variations of the total pools of *psbA* and *psbD* transcripts, encoding the two major subunits of the reaction center II (D1 and D2, respectively), closely matched the modulated fluctuations of growth irradiance, with a slight increase in the relative amplitude of the peak of *psbA* transcripts under UVR. It is worth noting that in the case of *Synechococcus*, which possesses four *psbA* genes (compared to only one in *P. marinus* MED4/PCC9511; Garczarek et al., 2008), this global expression pattern translates the temporal succession of the single copy gene encoding the D1.1 isoform (*SynWH7803_0784*) and the three copies encoding the D1.2 isoforms (*SynWH7803_0790*, *0366*, and *2084*), the former being repressed at noon and maximum at the LDT, while the three latter exhibited an opposite behavior (note that the expression levels of *SynWH7803_0366* and *2084* cannot be measured separately). Accordingly, the expression pattern of *PMM0743* and *SynWH7803_1216*, encoding the FtsH2 protease that is involved in the clearance of damaged D1 proteins from inactivated PSII (Komenda et al., 2006, 2010), showed a broad maximum during the period of highest photon fluxes (9:00–15:00) in both species. However, in *Prochlorococcus*, this gene showed a minimum at dusk, then a continuous increase during the dark period and the early morning, while in *Synechococcus* its relative expression levels were minimal all over the dark period from 18:00 to 6:00.

In contrast to reaction center II genes, the diel expression pattern of *psaB*, encoding one of the two major subunits of the PSI core, was very different between the two cyanobacteria. While in *Synechococcus* its expression level peaked between 15:00 and 18:00 and remained low for the rest of the L/D cycle, *Prochlorococcus* *psaB* transcript level was at its lowest around noontime and was maximal during the night period. Genes encoding light-harvesting systems also behaved quite differently between *Prochlorococcus* and *Synechococcus*. In the former organism, *pcbA* transcripts displayed a comparable pattern during the day to that of *psaB* transcripts with a significant downregulation in both light conditions, though the relative level of expression was even lower at noon for UV-irradiated cells. In contrast, for *Synechococcus* in VL, all genes encoding the different phycobiliprotein α-subunits (*apcA*, *rpcA*, *cpeA*, and *mpeA*) and phycoerythrin (PE) linkers (*cpeE*, *mpeD*, *mpeE*) were highly upregulated during the light period, with maximal expression at 15:00. While the expression patterns of genes coding for allophycocyanin, phycocyanin, and PEI subunits and their linkers were little affected by UVR, the expression of PEII genes showed a drop at noon. Interestingly, the *mpeE* gene, encoding the linker binding the distal PEII disk in this strain (Six et al., 2007c), even exhibited a 3-h delay of its maximal expression level in VL + UV compared to VL only.

Pigment biosynthesis genes also showed large variations of relative expression over the day under both light conditions. The *chlG* gene, encoding the chlorophyll *a* synthase, also behaved in an opposite way between *Prochlorococcus* and *Synechococcus*, with minimal and maximal expression levels at noon, respectively, in both cases more marked for UV-acclimated cells. Accordingly, the diel expression pattern of *PcCao*, a *Prochlorococcus*-specific gene encoding the chlorophyll *b* synthase (Satoh and Tanaka, 2006), also somewhat mirrored diel variations of growth irradiances. In contrast, the *ctrR* gene, encoding the β-Car hydroxylase that catalyzes the last biosynthesis step of zeaxanthin, usually considered as a photoprotective pigment, was maximally expressed during daytime in both organisms, but with a different global pattern, since in *Prochlorococcus* there was a strong surge in expression levels at 9:00 am followed by a progressive decline for the rest of the day, whereas in *Synechococcus* the diel pattern again closely matched the growth irradiance oscillations.

Most marine *Synechococcus* strains, including WH7803, contain the *ocp* gene, which encodes the orange carotenoid protein (OCP), a pigment-protein complex likely located between the phycobilisomes (PBS) and the PSII reaction center and involved in the dissipation of excess energy as heat (Boulay et al., 2008; Kirilovsky and Kerfeld, 2012). In VL, WH7803 *ocp* was maximally expressed during the day with a peak at 9:00, whereas the expression maximum was considerably higher and broadened toward noontime in UV-acclimated cells, suggesting a key role of this gene in the protection against UVR. Although all *Prochlorococcus* strains lack *ocp*, due to the absence of functional PBS, high light-adapted *Prochlorococcus* strains (including MED4/PCC9511), low-light-adapted ecotype LLI (Scanlan et al., 2009) as well as a few marine *Synechococcus* strains (though not WH7803) possess another potentially important gene for photoprotection, *ptox*, which encodes a plastid terminal oxidase. This enzyme was suggested to extract electrons from the electron transport chain between PSII and PSI and to re-oxide the plastoquinone pool reduced by PSII (Bailey et al., 2008; Berg et al., 2011). Interestingly, *ptox* exhibited very large variations in relative expression with a maximum at 15:00 (Figure 7), though contrary to *ocp*, its diel expression pattern was virtually identical between VL and VL + UV.

The ATPase genes *atpD* et *atpH* showed similar expression patterns in *Synechococcus* with high values from 9:00 to 15:00 in VL and even higher under VL + UV, whereas expression of these genes decreased all over the day in *Prochlorococcus* followed by a night recovery (Figure 7; see also Figure S3 in Kolowrat et al., 2010). An opposite behavior between the two strains was also observed for the *rbcL* gene, coding for the large subunit of RuBisCO (ribulose-1,5-bisphosphate carboxylase/oxygenase), the major enzyme of the carbon fixation process, except that in *Prochlorococcus*, there was a much sharper drop of expression levels than for *atp* genes at 15:00 in VL, and at 18:00 under VL + UV (Figure 7).

The strikingly different diel expression patterns observed between *Prochlorococcus* and *Synechococcus* for genes involved in light-harvesting, ATP formation, as well as the light-independent reactions of photosynthesis (Calvin–Benson cycle) suggest that most photosynthetic processes are probably controlled by distinct regulation networks in these two cyanobacteria, despite their close phylogenetic relatedness.

#### Redox and ROS detoxification

Most genes involved in the regulation of redox state and ROS detoxification pathways that are shared by *Synechococcus* sp. WH7803 and *P. marinus* PCC 9511 were differentially regulated during the L/D cycle in both picocyanobacteria and their diel expression patterns were comparable in VL and VL + UV conditions. However, the daily amplitudes of variations of ROS genes were often much larger in *Synechococcus* than in *Prochlorococcus*. Three of the most differentially expressed genes in the former organism, i.e., those coding for rubredoxin (*rub*), glutathione peroxidase (*SynWH7803_0151*), and a 2-cys peroxiredoxin (*2-Cys prx*), had their maximal daily expression around noontime, again suggesting a tight regulation by light, and their relative expression level significantly increased under UV. In contrast, in *Prochlorococcus*, orthologs of these three genes (*PMM0295*, *PMM1006*, and *PMM0856*, respectively) showed no clear diel pattern. Interestingly, for each cyanobacterium, the different peroxiredoxin gene copies exhibited diel patterns very distinct from one another.

*Synechococcus* sp. WH7803 and *P. marinus* PCC 9511 also possess specific sets of genes involved in ROS-scavenging systems. Among these strain-specific genes, it is worth noting that the catalase-peroxidase gene *katG* of *Synechococcus* was notably upregulated during the day, with a peak at 15:00, while *Prochlorococcus* *PMM0211*, a NADPH-dependent thioredoxin-disulfide reductase (NTR system) was by far the most differentially expressed ROS-scavenging gene of this organism, with a maximal expression at 18:00 in VL. At last, while the only *sod* gene present in *Prochlorococcus*, *sodN*, encoding a Ni-binding superoxide dismutase (SOD), was slightly downregulated at noon relative to nighttime in VL and VL + UV, *Synechococcus* *sodB*, encoding a Fe-binding SOD, was in contrast strongly upregulated during the light period under both conditions. The second *sod* gene of *Synechococcus*, *sodC* that encodes a Cu/Zn-binding SOD, showed only faint diel variations of its expression in VL, but was slightly downregulated at 20:00 in VL + UV.

#### Cell cycle, DNA repair, and circadian clock

In *Synechococcus* sp. WH7803, the DNA replication initiation factor gene *dnaA* was upregulated during the day with a broad peak of expression during the 9:00–15:00 time period in both light conditions. Subtle differences can however be noted between the two conditions, since the expression maximum seemingly occurred at 9:00 in VL and at noon in VL + UV (Figure 7). The *ftsZ* gene, which controls the biosynthesis of the Z-ring formed in the middle of the cell prior to cell division, showed very similar diel expression patterns to *dnaA*, except for systematically lower amplitudes of variations over the L/D cycle. The last cell cycle gene that was examined, *sepF*, which codes for a protein interacting with FtsZ during septum formation, showed a bell-shaped diel expression pattern peaking at 15:00 in both light conditions, though relative mRNA levels were again slightly higher in UV-acclimated cells.

In *P. marinus* PCC 9511 grown in VL, the *ftsZ* gene expression level peaked at 15:00, whereas *dnaA* and *sepF* both reached a maximum 3 h later. The relative expression levels of all three genes were considerably lower during the day when cells were grown under VL + UV, as previously reported (Kolowrat et al., 2010).

Since UVR are known to have deleterious effects on DNA structure, we also examined the diel expression patterns of a few genes involved in DNA repair pathways. The *recA* gene, which encodes an ATPase involved in the repair of double-strand breaks (DSBs) by homologous recombination (Chen et al., 2008), showed a broad maxima during daytime in *Synechococcus* cells grown in VL, while the relative expression of this gene was enhanced under VL + UV and remain high until 20:00 (Figure 7). In contrast, in *Prochlorococcus*, the *recA* expression peak occurred at the LDT in VL and was delayed by 2 h in the presence of UVR. The *uvrA* gene, encoding one subunit of the excinuclease UvrABC, an enzyme of the nucleotide excision DNA repair (NER) pathway, exhibited a sharp increase of its expression level during the lit period in both picocyanobacteria, with a peak at noon under both VL and VL + UV conditions. However, the maximal *uvrA* expression level was higher in UV-acclimated *Synechococcus* cells than in VL, whereas in *Prochlorococcus*, patterns were comparable between the two conditions (except at 9:00; Figure 7; Kolowrat et al., 2010).

Both picocyanobacteria also possess a family of genes related to DNA photolyases. In *Synechococcus*, the pattern of the photolyase gene *phrA* (Ng et al., 2000) closely matched the L/D cycle, with a peak at noon, which was threefold higher in VL + UV with regard to VL only (Figure 7). On the contrary, in *Prochlorococcus*, the *phrA* expression peak heights were similar in the two light conditions, even though the relative *phrA* mRNA level was higher at mid-morning in VL than VL + UV (Kolowrat et al., 2010). Like other marine *Synechococcus* but unlike *Prochlorococcus*, WH7803 possesses a second member of the DNA photolyase family (PhrB), which was recently suggested to be a cryptochrome (Goosen and Moolenaar, 2008). Interestingly, its diel expression pattern was quite similar to *phrA*, but with a reduced amplitude of variation of the relative mRNA amounts during daytime, even if there was again a very strong relative increase of the expression maximum under UV. The same was observed for a gene encoding an uncharacterized photolyase relative in *Synechococcus* (*SynWH7803*_*1271*), while its ortholog in *Prochlorococcus* (*PMM0425*) showed no clear diel pattern. Other, shorter members of the Phr family were also analyzed. Indeed, *Synechococcus* possesses a cluster of two genes (*SynWH7803_0266* and *0267*), each of which corresponds to one of the two domains of photolyases (photolyase and FAD-binding domains, respectively). So, once translated, they might potentially form an additional heterodimeric photolyase complex, as suggested by their diel pattern similar to *phrA* and high relative expression level (Figure 7). Interestingly, *Prochlorococcus* also possesses a gene (*PMM1360*) encoding a FAD-binding domain-containing protein, but surprisingly no gene coding for the other half of a photolyase (Scanlan et al., 2009; Partensky and Garczarek, 2010). Again, this gene had a very similar diel pattern as *phrA* from the same organism but with a much lower expression level.

The diel expression of genes involved into the circadian clock machinery was also studied given their central role in controlling global rhythmic transcriptional activity of the cells. While all marine *Synechococcus* possess the three *kai* genes coding for the core circadian oscillator, *Prochlorococcus* lacks *kaiA* and its circadian clock rather behaves like an “hourglass” that is reset every morning (Holtzendorff et al., 2008; Axmann et al., 2009). Interestingly, *kai* genes had a completely different behavior between the two cyanobacteria. In VL, all three *kai* genes of *Synechococcus* sp. WH7803 exhibited large diel variations with a broad maximum relative expression level at the end of the light period (15:00–18:00), while in *Prochlorococcus*, the expression of *kaiB* was minimal at noon, then increased continuously until dawn and there was no significant diel oscillation of *kaiC* mRNA. Presence of UVR caused a strong decrease of the level of expression of *kaiABC* genes at 15:00 in *Synechococcus* leading to a sharpening of their expression peaks, whereas in *Prochlorococcus*, the *kaiB* gene was further downregulated at noon without time shift. We also looked at the expression of *rpaA*, which codes for a DNA-binding protein acting as the response regulator of the KaiC-interacting kinase *SasA*, a two-component system that mediates the diel oscillations generated by KaiC phosphorylation to global transcription rhythms (Takai et al., 2006). RpaA is also known to be involved in the regulation of energy transfer from PBS to PSI and to interact with ferredoxin (Ashby and Mullineaux, 1999; Hanke et al., 2011). In both organisms, *rpaA* was maximally expressed around noontime and UVR had no significant effect on its diel transcription pattern.

Two genes encoding type II σ factors (*rpoD4* and *rpoD8*), that have been shown to modulate gene expression under different conditions and to respond to L/D stimuli (Imamura et al., 2003; Summerfield and Sherman, 2007), were also analyzed to assess their potential role in controlling global gene expression during L/D transitions. In *Synechococcus*, *rpoD4* exhibited a comparable diel expression pattern in VL and VL + UV, with a continuous increase during the day, a peak at the LDT then a progressive night decrease. In contrast, *rpoD8* showed only mild variations in VL while its diel pattern was prominent in UV-acclimated cells, with a strong upregulation during the day and a downregulation during the night. In *Prochlorococcus*, the pattern of these genes has been previously described (Kolowrat et al., 2010). Briefly, *rpoD8* has a low maximal expression in the mid-morning to noon period, whereas *rpoD4* peaked at the end of the light period (see also Figure 7).

At last, we looked at the expression of *nblS*, encoding a membrane-bound, sensor histidine kinase involved in the control by light of the expression of several photosynthesis-related genes, including all three *psbA* genes, the *cpcBA* operon, and some *hli* genes (van Waasbergen et al., 2002; Kappell et al., 2006). In VL, this gene was upregulated during the day with a peak in the morning and its maximum expression level increased almost twofold in the presence of UVR (Figure 7).

## Discussion

### Differences in photosystem activity and regulation between *Prochlorococcus* and *Synechococcus*

The alternation of light and darkness is one of the most predictive events that cyanobacteria have to deal with in the field. Strong variations of PAR occur over a daily timescale and are associated, in near surface waters, with concomitant changes of UVR fluxes. Here, we compare PCC9511, a strain representative of the *Prochlorococcus* HLI clade found in near surface oligotrophic waters, to WH7803, a *Synechococcus* strain characteristic of mesotrophic areas. Although these model strains do not represent the whole physiological diversity existing within these two genera, our data clearly show that both *Prochlorococcus* and *Synechococcus* cells are able to tune their photosynthetic apparatus to diurnal irradiance fluctuations. However, *Prochlorococcus* proved to be more sensitive than *Synechococcus* to photoinhibition by high photon fluxes, as suggested by a marked drop of the cellular pool of PSII core proteins (Figure 5) and a larger decrease of the PSII quantum yield (Figure 3) around noontime. It is noteworthy however that the latter phenomenon might also partly be due to NPQ of PSII fluorescence associated with photoprotective dissipation of light energy as heat (Bailey et al., 2005; Boulay et al., 2008). The diel changes of the photosynthetic activity observed here for *P. marinus* PCC 9511 (Figure 3) are quite comparable with those previously described for this strain grown in similar light conditions (Bruyant et al., 2005), except that our cultures exhibited a higher *F*_V_/*F*_M_ during the night (∼0.7 vs. ∼0.55), likely translating a slightly better physiological status. However, they contrast with those obtained on the closely related MED4 strain by Zinser et al. (2009), who did not observed any significant diel variation of the *F*_V_/*F*_M_, likely because it was grown at lower irradiance (∼230 vs. 870 μmol photon m^−2^ s^−1^ here) and over a different L/D cycle (14/10 vs. 12/12 h). Another noticeable observation from our study was that UVR did not cause any appreciably stronger photoinhibitory effect than VL in both genera, likely due to an increased repair rate under VL + UV compared to VL. However, this UV-induced repair was much more important for *Synechococcus* than *Prochlorococcus* (∼twofold vs. ∼fivefold at noon, respectively). Accordingly, in the former organism, the relative D1 content was enhanced in the presence of UVR, while in *Prochlorococcus* the noontime drop of D1 was somewhat extended for UV-acclimated cells (see also transcriptomic analyses of the *psbA* genes below). These results are consistent with those obtained by Six et al. (2007a), who observed that in response to a transient high light exposure, *P. marinus* PCC 9511 exhibited a lower PSII repair rate (0.9 PSII gained per second) than a range of marine *Synechococcus* strains (1.1–1.6 PSII s^−1^). In both strains in VL, this rate was more or less proportional to the instantaneous irradiance. However for cells grown under VL + UV, while the repair rate was comparable between the two strains at 9:00 and 15:00, it was significantly depressed at noon in *Prochlorococcus* compared to *Synechococcus*. This suggests that the PSII repair capacity of *Prochlorococcus* was already maximum around 400 μmol photons m^−2^ s^−1^ (Figure 4B).

The occurrence of different D1 encoding gene copies in *Prochlorococcus* and *Synechococcus* could, at least partially, explain such a distinct behavior. Indeed, while *Prochlorococcus* strains have one to three identical *psbA* gene copies (one in PCC 9511), coding for a single D1:1-like isoform (Hess et al., 1995; Partensky and Garczarek, 2003), *Synechococcus* strains possess three to six *psbA* genes, with only one copy coding for a D1:1 isoform and two to five copies (three in WH7803), coding for D1:2 isoforms (Garczarek et al., 2008). The respective role of these isoforms has been widely studied in the literature, both in freshwater cyanobacteria (Bustos et al., 1990; Clarke et al., 1993; Campbell et al., 1995, 1998a; Sass et al., 1997; Kos et al., 2008) and in marine picocyanobacteria (Garcia-Fernandez et al., 1998; Garczarek et al., 2008). Although some variations among cyanobacteria have been observed, it is generally accepted that the D1:1 isoform would confer a higher PSII activity (Campbell et al., 1996), while D1:2 would provide a lower quantum yield but a higher PSII resistance to photoinhibition (Krupa et al., 1991;Campbell et al., 1995, 1998a; Tichy et al., 2003). A variety of environmental cues, including UV exposure, can induce the exchange of these isoforms (Sicora et al., 2006, 2008; Garczarek et al., 2008; for a review, see Bouchard et al., 2006). Here, we indeed noticed in *Synechococcus* sp. WH7803 an opposite expression pattern of D1:1 (*SynWH7803_*0784) and D1:2 isoforms encoding genes (*SynWH7803_0790*, *0366*, and *2084*) during daytime (Figure 7). It is worth noting that while there were only slight discrepancies in the expression levels of the different D1:2 encoding genes between VL and VL + UV, the D1:1 encoding gene was about fivefold more repressed at noon under the latter condition, suggesting a complete replacement of the D1:1 isoform by D1:2 isoform(s) that may contribute to the slight midday decrease in PSII quantum yield (Figure 3). Interestingly, although the single isoform in *Prochlorococcus* is phylogenetically a D1:1 isoform, as confirmed by the occurrence of a Gln residue at position 130 of the amino acid sequence (instead of Glu in D1:2; Clarke et al., 1993; Giorgi et al., 1996), its transcriptomic pattern was clearly closer from that of D1:2 isoforms, at least in our culture conditions. It is likely however that it presents a lower resistance to PSII photoinactivation compared to a true D1:2, as suggested by the larger drop of *F*_V_/*F*_M_ at noon (Figure 3) and the concomitant lower PSII repair rate (Figure 4), compared to *Synechococcus*.

Interestingly, the D2 subunit of the PSII core is also encoded by a single *psbD* gene in all *Prochlorococcus* genomes, whereas all *Synechococcus* sequenced so far possess two nearly identical *psbD* genes, as in most other cyanobacteria (such as e.g., *Synechococcus* sp. PCC 7942; Golden et al., 1989), with one co-transcribed with *psbC* that encodes the internal PSII antenna protein CP43 (Garczarek et al., 2001) and the other isolated in the genome. It is likely that as for *psbA*, the two copies are differently regulated in response to light and/or UV stress as previously reported in freshwater model cyanobacteria (Bustos and Golden, 1992; Kos et al., 2008), although this was not checked in the present study. Alternatively, this may simply contribute to a higher expression level of this key photosynthetic gene, possibly enabling a higher turnover of the corresponding protein.

Another strategy used by cyanobacteria to cope with excess light energy is to decrease the relative amount of PSI reaction center complexes, an adjustment that was shown to decelerate the rate of photosynthetic electron transport (Murakami and Fujita, 1991; Hihara et al., 1998; Muramatsu and Hihara, 2003). Indeed, experiments on *Synechocystis* sp. PCC 6803 mutants impaired in their ability to modulate photosystem stoichiometry showed that this capacity is indispensable for growth under continuous high irradiance (Hihara et al., 1998; Fujimori et al., 2005). Accordingly, in *Synechococcus* sp. WH7803 the relative *psaB* levels were low before dawn (Figure 7), likely reflecting a lower PSI cell content at this time of the day, as previously observed in *Crocosphaera* *watsonii* (Saito et al., 2011). In contrast, this was not the case in *Prochlorococcus*, in which PSI core transcripts were maximal over most of the dark period.

### Differential regulation of light-harvesting systems in response to high light and UV radiations

The striking structural differences between the major PSII antenna complexes of *Prochlorococcus* and *Synechococcus* may also be partially responsible for the different sensitivity of these picocyanobacteria to UV stress. Indeed, whilst *Synechococcus*, as most cyanobacteria, possess a large membrane-extrinsic antenna, the PBS (Sidler, 1994; Six et al., 2007c), *Prochlorococcus*, like the other two green oxyphotobacteria, *Prochloron* and *Prochlorothrix*, use a transmembrane Chl *a/b*-binding Pcb antenna (LaRoche et al., 1996; Garczarek et al., 2003; Partensky and Garczarek, 2003). These dissimilar antenna structures may have important consequences on the way excitation energy is funneled downhill toward the reaction centers, on the regulation of this process as well as on the amount and nature of damages caused by UVR on *Prochlorococcus* and *Synechococcus* cells, since both organisms show some absorption capacities in the 300- to 400-nm band, which are likely related to their antenna (see e.g., Ong and Glazer, 1991; Claustre et al., 2002). Comparing and monitoring UV cross-sections for both picocyanobacteria would help answering this question.

**Figure 8:**
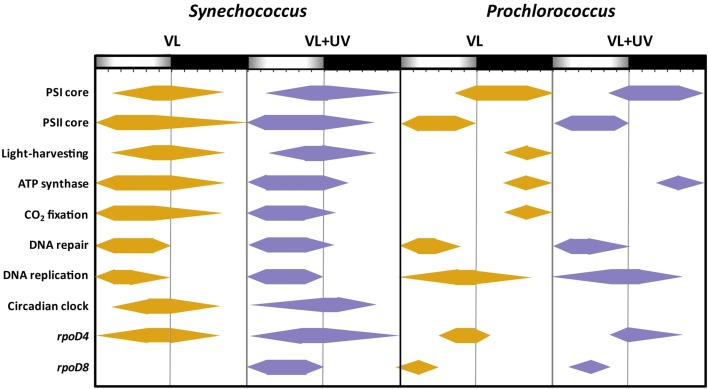
**Scheme of the daily patterns of gene upregulation for several important functional for picocyanobacterial cells acclimated to a modulated 12/12 h L/D cycle of high visible light with or without UV radiations**. This figure, derived from Figure 7, shows for each individual the time intervals during which they were significantly upregulated [|log_2_ (Fold Change)| > 1.0], with regard to the expression level measured at 6:00 in VL.

**Figure A1:**
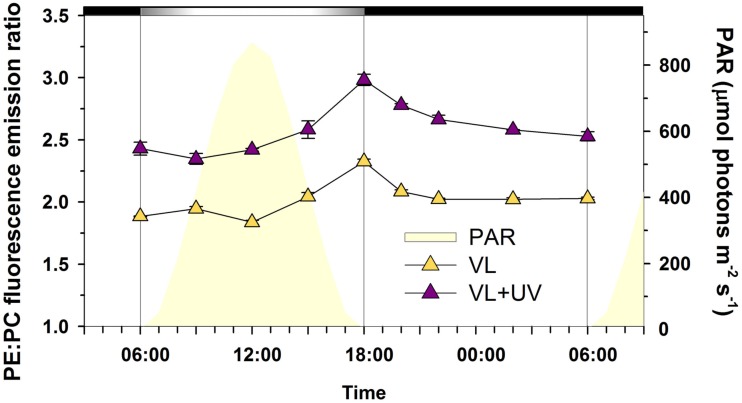
**Phycoerythrin to phycocyanin (PE:PC) fluorescence emission ratio as measured by spectrofluorimetry (at 572 and 651 nm, respectively) in *Synechococcus* sp. WH7803 cells grown under a L/D cycle with and without UV**. This ratio showed significantly higher values in VL + UV than in VL-acclimated cells. In both light conditions, it increased during the day by ∼25% compared to the value at dawn then decreased during the night. The amplitude of these oscillations (ca 0.4 unit) was very low compared to those reported for *Synechococcus* sp. WH8102 acclimated to a large range of irradiance (2.5 units; Six et al., 2004). Further investigation is required to determine whether the diel pattern of PE:PC fluorescence emission ratio rather results from a decrease in the efficiency of energy transfer in the PBS rod and/or a progressive accumulation of free phycoerythrins during the day. These light-induced oscillations might be indirectly related with state transitions (Campbell et al., 1998b; Mullineaux and Emlyn-Jones, 2005), a light-dependent process which usually exhibits large amplitudes in marine *Synechococcus* (Six et al., personal communication).

Here, we indeed observed an opposite expression pattern between the genes encoding antenna systems from these two organisms, with a strong downregulation of the *pcb* gene in *Prochlorococcus* during day time under VL (see also Garczarek et al., 2001) that was even more dramatic under VL + UV, while all examined PBS genes were upregulated in the afternoon in both light conditions (Figure 8). A similar result was previously obtained in L/D-entrained *Cyanothece* sp. ATCC 51142 cultures, though in the latter case maximum expression was centered at midday (Toepel et al., 2008). These observations suggest that the biosynthesis of antenna complexes occurs at different times of the day in the two picocyanobacteria and is under different light regulation controls. The fact that PBS genes remained upregulated under VL + UV suggests that the buildup of PBS complexes was only moderately affected by these radiations. This contrasts with a previous study where cultures of *Synechococcus* sp. WH8102 grown in continuous low-light were subjected to a sudden shift to UV (Six et al., 2007b). This stress provoked a strong decrease in the relative expression of all PBS genes, associated with a disconnection of the PBS complexes from the thylakoid membrane as well as a dissociation of the distal PEII disks of PBS rods. In the present study, where cells were acclimated to either VL or VL + UV for several weeks, the PE to PC fluorescence emission ratio did not exhibit a peak at noon in either light condition, as would be expected if terminal PE subunits (i.e., PEII) were disrupted (Figure A1 in Appendix). The higher values of this ratio in UV-acclimated *Synechococcus* cells are likely not related to a higher PE content of the PBS, but rather to permanently decoupled PEII subunits, which would dissipate incident light as fluorescence. Consistently, UV also induced a slight drop of the expression of most PEII genes at noon and a 3-h delay in the timing of the expression peak of *mpeE*, which encodes the linker binding the terminal PEII disk in *Synechococcus* sp. WH7803 (Six et al., 2007c).

### Differential photoprotection mechanisms

*Prochlorococcus* and *Synechococcus* have also developed specific mechanisms to dissipate excess light energy. Most *Synechococcus* strains possess one gene encoding the orange carotenoid protein (OCP; for a recent review, see Kirilovsky and Kerfeld, 2012), which is thought to mediate energy dissipation as heat through interaction with the PBS core, thus inducing a NPQ of PSII fluorescence. Here, we observed that the *ocp* gene was upregulated during daytime in VL and that UVR dramatically enhanced its expression in the early light period that may trigger a temporary increase of the OCP cellular pool and/or activity. To our knowledge, this is a first time that UV is shown to control the expression of this gene since it is usually believed that OCP is only blue light sensitive (Wilson et al., 2008; Kirilovsky and Kerfeld, 2012).

While all *Prochlorococcus* strains lack OCP, all high light-adapted strains (including PCC 9511) and some low-light-adapted strains possess an homolog of *PTOX*, which is thought to extract electrons between PSII and PSI and to combine them with protons and oxygen to generate water (Bailey et al., 2005, 2008). The *ptox* gene was (with *ftsZ*) one of the two most differentially expressed genes among those analyzed here in PCC 9511, with a maximal relative fold change over the day of ∼73-fold [or log2(FC) = 6.2] at 15:00. These high values are consistent with previous results on the closely related strain *P. marinus* MED4 (Zinser et al., 2009; Berg et al., 2011). So the alternative electron flow to oxygen triggered by PTOX might be an important mechanism used by *Prochlorococcus* to struggle against excess light energy arising to PSII (Bailey et al., 2008; Berg et al., 2011), although high light induced proteins (HLIPs), which were not analyzed in the present study, are also likely important contributors in this process (He et al., 2001).

Other key actors in photoprotection mechanisms are carotenoids. While the cellular localization and precise action mechanism of Zea is unclear yet, Car are known to be mostly bound to PS reaction centers, two of them in close vicinity of Chls, and help mitigate oxidative damages, mainly by quenching the ^1^O_2_ species resulting from the de-excitation of Chl triplets (Telfer, 2005). Both Zea:Chl *a* and Car:Chl *a* ratios appeared to be tightly coupled to the L/D cycle in *Synechococcus*, but less so in *Prochlorococcus*, for which we observed a continuous increase of both ratios during most of the day and a symmetrical drop during the night concomitant with cell division, as previously noticed by Claustre et al. (2002). In *Synechococcus*, the sharp drop of the β-Car:Chl *a* ratio from noon to dusk, which was more pronounced in UV-exposed cells (Figure 2C), likely reflects the progressive destruction of β-Car molecules by oxidative stress (Telfer, 2005). β-Car was then seemingly regenerated at low rate during the night, then much more rapidly during the first hours of the day, suggesting a light-dependency of the β-Car biosynthesis process, as previously reported in other cyanobacteria (Steiger et al., 2005; Ryu et al., 2010). Similarly, the light-modulated variations of the Zea:Chl *a* ratio during the day in *Synechococcus* in both light conditions could be due either to a higher degradation rate of Chl *a* relative to Zea or to a higher synthesis rate of the latter pigment, a likely photoprotective mechanism against high midday photon fluxes. The observed strong upregulation of *crtR* at high irradiances tend to favor the second hypothesis (Figure 7). A further increase of the relative *crtR* transcript levels at noon in VL + UV, which was not translated into a significantly higher midday increase in the Zea:Chl *a* ratio than in VL (Figure 3A), suggests that Zea msolecules might have a particularly high turnover under UV. In both genera, the systematically higher values of Zea:Chl *a* ratio under VL + UV than VL only suggest that UV exposure induces a comparable response as a long-term acclimation to high irradiance, as previously reported in other marine cyanobacterial strains (Kana and Glibert, 1987; Moore et al., 1995; Six et al., 2004). Altogether, our results suggest the occurrence of a light-controlled anabolism/catabolism cycle of both photoprotectants (Car and Zea) in *Synechococcus*, while diel changes in the pigment content of *Prochlorococcus* cells were rather related to the cell division cycle or other factors not analyzed here such as PS stoichiometry and/or antenna size.

### Differential protection mechanisms against reactive oxygen species-induced damages

Light-harvesting complexes are not only sources but also major targets of ROS (Sies and Menck, 1992; Krieger-Liszkay, 2005). Because of their localization within the thylakoid membrane around PSII complexes (Bibby et al., 2003), the divinyl-Chl *a*/*b*-binding Pcb antennae of *P. marinus* PCC 9511 likely produce ROS, particularly noxious for this photosystem. Indeed, it has been shown in other Chl *b*-containing organisms that excited singlet Chls lead to the synthesis of Chl triplet states that can react with ^3^O_2_ to produce the very reactive species^1^O_2_ (Sies and Menck, 1992; Krieger-Liszkay, 2005). Assays performed here by directly adding H_2_O_2_ to sub-cultures at different time points of the modulated L/D cycle strongly suggest that the effect of ROS increased in a light-dependent manner for both organisms. Consistently, a synergistic effect of light and oxidative stress on PSII photoinactivation was previously demonstrated by Blot et al. (2011) who showed in *Synechococcus* sp. WH7803 that ROS may induce PSII inactivation through both direct damages to the reaction center II and inhibition of the PSII repair cycle. The latter phenomenon resulted in faster PSII inactivation in high light- than in low-light-acclimated cultures, due to their higher D1 turnover. Furthermore, *Prochlorococcus* was also more sensitive to H_2_O_2_-triggered stress than *Synechococcus* during the night, which might be related to a lower intrinsic resistance of its PSII.

The difference in ROS sensitivity between *Prochlorococcus* and *Synechococcus* strains might be explained, at least partially, by a more complete set of genes involved in ROS protection and detoxification in *Synechococcus*. Indeed, most *Prochlorococcus* lineages have experienced an extensive genome streamlining during evolution, resulting in the loss of a large number of non-essential but potentially useful genes in this context (Figure 7; Dufresne et al., 2005; Scanlan et al., 2009; Partensky and Garczarek, 2010). For instance, while *Synechococcus* sp. WH7803 synthesizes one catalase/peroxidase (KatG), *Prochlorococcus* has neither catalases nor peroxidases. *Prochlorococcus* also lack the *ftrC* and *ftrV* genes, encoding the two subunits of ferredoxin-thioredoxin reductase (FTR), as well as one ferredoxin and one thioredoxin, potentially associated to this complex (Dufresne et al., 2008). Furthermore, while *Synechococcus* possesses two superoxide dismutase (SOD), one Fe-type and one Cu-Zn-type, *Prochlorococcus* has only one, Ni-type SOD (SodN; Scanlan et al., 2009). In the present study, the expression levels of most ROS genes showed strikingly higher amplitudes of variation over the day in *Synechococcus* than in *Prochlorococcus*. Even though transcriptomic data are not necessarily synchronized with the activity of the corresponding enzymes, the lower resistance of *Prochlorococcus* to high VL and UVR could at least partially be due to a higher sensitivity to light-driven oxidative stress.

## Conclusion

The comparison of *Synechococcus* and *Prochlorococcus* cultures acclimated to VL supplemented or not with UVR revealed relatively few physiological responses specific to UV. This notably includes a shift of the DNA synthesis phase (Figure 1; see also Kolowrat et al., 2010), an increase of the Zea:Chl *a* ratio (Figure 2) and an enhanced PSII repair rate (Figure 4). Accordingly, UVR seemingly also had limited effects at the transcriptomic level, as shown by the globally similar diel expression patterns between VL and VL + UV in both strains (Figures 7 and 8). It is worth noting however that the relative expression of a few genes was either enhanced (e.g., *psbD* in both strains, D1:2 encoding genes and *crtR* in *Synechococcus* only) or reduced (e.g., *Synechococcus* D1:1 encoding gene or *Prochlorococcus pcbA*) in UV-acclimated cultures. A handful of genes, including *rpoD4*/8 in *Prochlorococcus* and *kaiABC* in *Synechococcus*, also exhibited a delayed expression peak by about 3 h. However, this surprisingly did not translate into any conspicuous changes in the diel patterns of most of the other genes examined here, as could have been expected from the known regulatory role of the circadian clock and sigma factors on gene transcription (see e.g., Summerfield and Sherman, 2007; Ito et al., 2009).

The most striking result of the present study is likely the markedly distinct response to diurnal light variations between *Prochlorococcus* and *Synechococcus*, despite their close phylogenetic relatedness (Scanlan et al., 2009). These two picocyanobacteria indeed exhibited very different degrees of PSII photoinactivation at noon that can be partly explained by their distinct (i) PSII repair capacity (Figure 4; see also Six et al., 2007a), (ii) ability to modulate photoprotective pigments (Zea and Car, Figure 2), and (iii) resistance capacity against oxidative stress (Figure 6). Comparative transcriptomic analyses also revealed that, in *Synechococcus*, genes coding for a number of protective systems were maximally expressed during hours of highest irradiance, i.e., when these mechanisms are most critical to cope with transitory stressful conditions. This includes several genes involved in ROS detoxification enzymes, DNA repair genes as well as genes involved in photoprotection and/or dissipation of excess energy, such as *crtR* and *ocp* (Figure 7). In contrast, in *Prochlorococcus*, very few genes of these pathways, including *ptox* and *psbA*, were maximally expressed around midday, while others, such as *phrA* and *crtR*, had already reached their maximal (saturating) expression at mid-morning. Furthermore, many photosynthetic genes that were upregulated during the day in *Synechococcus* were in contrast downregulated in *Prochlorococcus*, including the PSI core gene *psaB* as well as genes involved in light-harvesting, ATP synthase and CO_2_ fixation (Figure 8). Similarly, *glgA* (encoding glycogen synthase) mRNA abundance was recently shown to exhibit a maximum diel expression at midday in *Synechococcus* sp. WH8103 grown under a 16/8-h L/D cycle, while it peaked in concert with *rbcLS* in *Prochlorococcus* *marinus* MED4 during the LDT (Wyman and Thom, 2012). Thus altogether, it seems that while *Synechococcus*, as many other cyanobacteria (Kucho et al., 2004, 2005; Stockel et al., 2008; Toepel et al., 2008, 2009; Shi et al., 2010), efficiently copes with the diurnal changes in photon fluxes, *Prochlorococcus* rather displays a stress-like response at midday. The latter response is most likely related to the high irradiance level used in the present study (reaching 870 μmol photons m^−2^ s^−1^ at noon), which are typically found in the upper mixed layer of tropical oligotrophic oceans (Holtzendorff et al., 2001). Indeed, several genes that were found here to be downregulated during the light period (e.g., *pcb*, *rbcL*, *chlG*) were in contrast upregulated in *Prochlorococcus* cultures grown at lower irradiances, provided as continuous (Berg et al., 2011) or cyclic light (Zinser et al., 2009). Despite the very atypical transcriptomic response observed here, *Prochlorococcus* was able to recover high PSII quantum yield at night and to maintain an optimal growth rate under these conditions. Thus, we hypothesize that, in contrast to *Synechococcus* sp. WH7803, *P. marinus* PCC 9511 cells manage to cope with harmful light conditions by bringing down temporarily some of the main metabolic processes and by launching a minimal set of protection mechanisms during stressful hours. Whether the absence of a true circadian clock in *Prochlorococcus* (Holtzendorff et al., 2008; Axmann et al., 2009) is involved in this differential management of excess light compared to *Synechococcus* still remains to be investigated.

Altogether, our study reinforces previous studies depicting *Prochlorococcus* as a very specialized organism restricted to a narrow environmental niche, while *Synechococcus* has adopted a generalist strategy enabling it to cope with more variable environmental conditions, a difference consistent with the distinct habitats in which these two organisms predominate (Scanlan, 2003; Kettler et al., 2007; Dufresne et al., 2008; Scanlan et al., 2009).

## Conflict of Interest Statement

The authors declare that the research was conducted in the absence of any commercial or financial relationships that could be construed as a potential conflict of interest.

## Supplementary Material

The Supplementary Material for this article can be found online at http://www.frontiersin.org/Aquatic_Microbiology/10.3389/fmicb.2012.00285/abstract

Supplementary Table S1 **List of primers used for real time PCR reactions**. Most primers are targeting a specific gene. Exceptions include the *psbD pool* and *psbA pool* primers, which were designed to amplify the two *psbD* gene copies and all four *psbA* copies present in *Synechococcus* sp. WH7803, respectively. Furthermore, one primer set targets two *psbA* genes (*SynWH7803_0366* and *2084*), which have nearly identical coding sequence and 5′-UTR. Genes are classified by functional categories, as indicated in the first column. The Cyanorak database of picocyanobacteria protein families is publicly accessible at http://www.sb-roscoff.fr/Phyto/cyanorak/

Supplementary Table S2 **Daily variations of the expression of selected genes, as measured by real time quantitative PCR, for picocyanobacterial cells acclimated to a modulated 12/12 h L/D cycle of VL with or without UV radiations**. All data are expressed as log_2_ (Fold Change) ± mean deviation for two biological replicates. For each gene, transcript levels are normalized to the reference time point 6:00 in VL. Genes are classified by functional categories, as indicated in the first column. This table is a detailed version of Figure 7 of the main manuscript.
